# Association of TCTP with Centrosome and Microtubules

**DOI:** 10.1155/2012/541906

**Published:** 2012-05-14

**Authors:** Mariusz K. Jaglarz, Franck Bazile, Katarzyna Laskowska, Zbigniew Polanski, Franck Chesnel, Ewa Borsuk, Malgorzata Kloc, Jacek Z. Kubiak

**Affiliations:** ^1^Department of Developmental Biology & Invertebrate Morphology, Institute of Zoology, Jagiellonian University, 30-387 Krakow, Poland; ^2^CNRS, UMR 6290, Institut de Génétique et Développement de Rennes, Cell Cycle Group, 35043 Rennes, France; ^3^UEB, IFR 140, Faculté de Médecine, Université Rennes 1, 35043 Rennes, France; ^4^Department of Genetics & Evolution, Institute of Zoology, Jagiellonian University, 30-387 Krakow, Poland; ^5^Department of Embryology, Institute of Zoology, University of Warsaw, 02-096 Warszawa, Poland; ^6^Department of Surgery, The Methodist Hospital, Houston, TX 77030, USA; ^7^Immunobiology Laboratory, The Methodist Hospital Research Institute, Houston, TX 77030, USA

## Abstract

Translationally Controlled Tumour Protein (TCTP) associates with microtubules (MT), however, the details of this association are unknown. Here we analyze the relationship of TCTP with MTs and centrosomes in *Xenopus laevis* and mammalian cells using immunofluorescence, tagged TCTP expression and immunoelectron microscopy. We show that TCTP associates both with MTs and centrosomes at spindle poles when detected by species-specific antibodies and by Myc-XlTCTP expression in *Xenopus* and mammalian cells. However, when the antibodies against XlTCTP were used in mammalian cells, TCTP was detected exclusively in the centrosomes. These results suggest that a distinct pool of TCTP may be specific for, and associate with, the centrosomes. Double labelling for TCTP and *γ*-tubulin with immuno-gold electron microscopy in *Xenopus laevis* oogonia shows localization of TCTP at the periphery of the *γ*-tubulin-containing pericentriolar material (PCM) enveloping the centriole. TCTP localizes in the close vicinity of, but not directly on the MTs in *Xenopus* ovary suggesting that this association requires unidentified linker proteins. Thus, we show for the first time: (1) the association of TCTP with centrosomes, (2) peripheral localization of TCTP in relation to the centriole and the *γ*-tubulin-containing PCM within the centrosome, and (3) the indirect association of TCTP with MTs.

## 1. Introduction

Translationally Controlled Tumour Protein (TCTP) is implicated in a broad diversity of cellular functions. It stimulates cell proliferation, growth, survival, and stress response [1]. It is very abundant in highly proliferating cells, including cancer cells. The interest in TCTP increased rapidly in recent years because of the growing body of evidence for its key role in carcinogenesis and rare phenomenon of tumour reversion [2, 3]. Recently, it was elegantly demonstrated that TCTP expression is negatively regulated by p53 and *vice versa*, that is, TCTP negatively regulates p53 cellular levels via induction of its degradation triggered by MDM2 ubiquitin ligase [4]. The evidence of the reciprocal feedback between TCTP and p53 gives additional proof of the importance of TCTP in cancer development and progression/reversion. TCTP is also associated with the cytoskeleton and throughout this association impacts cell shape, motility, metastasis, and the aggressiveness of cancer. It has been established that TCTP associates both with actin microfilaments (MFs) and MTs [5]. Biochemical analysis of these interactions suggested that, most likely, the TCTP interacts with MFs and MTs indirectly; however, details of these interactions remain unknown (ibid.). TCTP knock down modifies drastically the cell shape and both MFs and MTs architecture [5, 6]. TCTP acts in competition with actin-binding protein cofilin [7]. Because the cofilin promotes actin disassembly, the competition with TCTP may result in increased actin polymerization in cells with higher TCTP levels. Much less is known about the relationship between TCTP and MTs. We have shown that TCTP and tubulin localization in *Xenopus* and human cells are very similar, but not identical suggesting a presence of “TCTP fibers” unrelated to MTs as well as the presence of TCTP-negative MTs [5]. TCTP localization within the mitotic spindle also does not overlap tubulin localization—it has more homogenous pattern, which suggests that either only a subpopulation of TCTP is associated with MTs or that TCTP localizes in the vicinity but not directly on MTs. On the other hand, TCTP seems to be very strongly associated with the poles of the spindle [5]. These observations suggested that TCTP may be associated with MTs via intermediate linker proteins and that TCTP may also be centrosome-associated protein. We investigated these hypotheses in the study presented here.

## 2. Material and Methods

### 2.1. Tissue Culture Cells

The XL2 cell line was cultured in L-15 medium supplemented with 10% fetal calf serum (FCS; full medium) and incubated at 25°C in air. HeLa, NIH3T3, and Cos7 cells were maintained in Dulbecco's modified Eagle's medium supplemented with 10% fetal calf serum (FCS) and incubated at 37°C in 5% CO_2_. Media were supplemented with penicillin (100 Units/mL) and streptomycin (100 mg/mL).

### 2.2. Immunocytochemistry

Cells seeded on glass coverslips were fixed in 75% methanol, 3.7% formaldehyde, 0.5x PBS, or in 3.7% paraformaldehyde in 1x PBS for 10 min at room temperature and permeabilized with 0.1% Triton X100 in PBS for 5 min. DNA was visualized using DAPI. Polyclonal antibodies against XlTCTP (produced in the laboratory in Rennes) and against HsTCTP (Santa-Cruz) or rat TCTP were used at the dilution of 1 : 1000 and 1 : 100, respectively, with overnight incubations at 4°C. Anti-*α*-tubulin (Sigma) and anti-*β*-tubulin (Euromedex) were diluted 1 : 200. Purified anti-c-myc antibody (Sigma) was diluted 1 : 100. Secondary antibodies (RITC conjugated, 1 : 1000 dilution; Molecular Probes) were incubated for 1 hr at room temperature. Coverslips were mounted in Vectashield and examined using a Leica DMRXA2 fluorescence microscope or Leica Confocal SP2 microscope. Photographs were taken using a black and white COOLsnap ES camera (Roper Scientific), and images were processed using Metamorph software (Universal Imaging).

### 2.3. Cell-Free Extracts and *In Vitro* Spindle Assembly

Cytostatic factor-arrested extracts (CSF extracts) were prepared as described by Murray [8]. For* in vitro* spindle assembly, 0.5 *μ*L of rhodamine-labeled bovine brain tubulin (Cytoskeleton) was added at 0.2 mg/mL and 2 *μ*L of sperm heads at a concentration of ~1000 nuclei/*μ*L added to 50 *μ*L of the extract and incubated for 60–90 min at 21°C. Spindles (15 *μ*L of extract) were prefixed in 1 mL BRB80 buffer (80 mM K-Pipes, pH 6.8, 1 mM EGTA, and 1 mM MgCl_2_) containing 30% glycerol, 1% paraformaldehyde, and 0.5% Triton X-100 and centrifuged (2300 xg, 30 min at room temperature) through a 40% glycerol cushion in BRB80 onto glass coverslips in 12-well plate. They were fixed by adding 1 mL cold methanol (−20°C) for 10 min at room temperature (isolated spindles). Then fixed spindles were processed for immunocytochemistry for TCTP using anti-XlTCTP, viewed, and photographed as the cells above.

### 2.4. Cell Transfection

For transfection of XL2 and NIH3T3 cells with plasmids encoding* Xenopus* Myc-TCTP, 5 × 10^5^ cells were plated on glass coverslips in a 12-well plate. Cells were transfected with 0.5 *μ*g of plasmid DNA using FuGENE 6 transfection reagent (ROCHE) following the manufacturer's instructions.

### 2.5. Mouse Oocytes

Three-month-old Swiss albino females were injected intraperitoneally with 10 IU pregnant mare serum gonadotrophin (PMSG; Folligon, Intervet, Holland) to stimulate the development of ovarian follicles. Forty eight to fifty two hours later females were killed by cervical dislocation. Fully grown oocytes arrested at prophase of the first meiotic division—germinal vesicle stage (GV) were released from ovarian follicles. Oocytes were freed from cumulus cells by pipetting and then cultured for 2 h in M2 medium containing bovine serum albumin (BSA; 4 mg/mL). Oocytes that resumed meiosis, that is, underwent germinal vesicle breakdown (GVBD) within first 2 h of *in vitro* culture, were used for further manipulations and collected for the following stages: GVBD, MI, (6 hrs after GVBD), and MII (20 hrs after GVBD). Oocytes were fixed in 3.7% formaldehyde in PBS, permeabilized with 0.01 Triton X100 in PBS, and subjected to immunofluorescence after incubation in the presence of XlTCTP antibody, the same as with the tissue culture cells.

### 2.6. Xenopus Laevis Tadpole Ovaries and Electron Microscopy

The developing ovaries were removed from anaesthetized tailed and tailless froglets (stages 62–66) of wild-type *Xenopus laevis*. Ovaries were fixed in TEM fixative (2% formaldehyde, 3% glutaraldehyde, EM grade, Ted Pella, Redding, CA, in 0.1 M sodium cacodylate buffer pH 7.3, Polysciences, Warrington, PA) containing 10 *μ*m taxol (Cytoskeleton, Denver, CO) to stabilize the microtubules and centrioles. To enhance the visualization of centrioles, the material was stained in 0.5% uranyl acetate, and the osmium tetraoxide treatment was omitted. This resulted in very light staining of all membranous structures; however, it allowed the visualization of highly contrasted centrioles and microtubules. Embedding and sectioning were done as described by Kloc et al. [9]. Postembedding immunostaining using anti-XlTCTP and anti-*γ*-tubulin antibodies was performed as described in Bilinski et al. [10]. For immunogold labeling, the ovaries were fixed as above. Ultrathin sections (60 nm thick) were collected on nickel single-slot grids (coated with formvar) and blocked with 2% bovine serum albumin (BSA; Sigma) in PBS and 0.1% NaN3 for 30 min, after overnight incubation at 4°C with the primary antibodies (rabbit anti-TCTP, or mouse monoclonal anti-*γ*-tubulin [GTU-88], ab11316, Abcam) diluted 1 : 50−1 : 100 in the incubation solution (PBS, 1% BSA, 0.1% NaN3). Following several washes in PBS, the grids were incubated for two hours, at room temperature, with the secondary antibody (goat anti-rabbit conjugated to 18 nm gold particles or goat anti-mouse conjugated to 10 nm gold particles, Jackson ImmunoResearch Lab.) diluted 1 : 100–1 : 200 in the incubation solution. Subsequently, the grids were washed in PBS and finally in distilled water. After drying, the sections were contrasted with uranyl acetate and lead citrate and viewed with a JEOL 100SX electron microscope at 80 kV. In control experiments, sections were treated exactly the same as described above, but there was no incubation with the primary antibody. The secondary antibodies were also tested for cross-reactivity prior to double labeling experiments.

## 3. Results and Discussion

**Figure 1:**
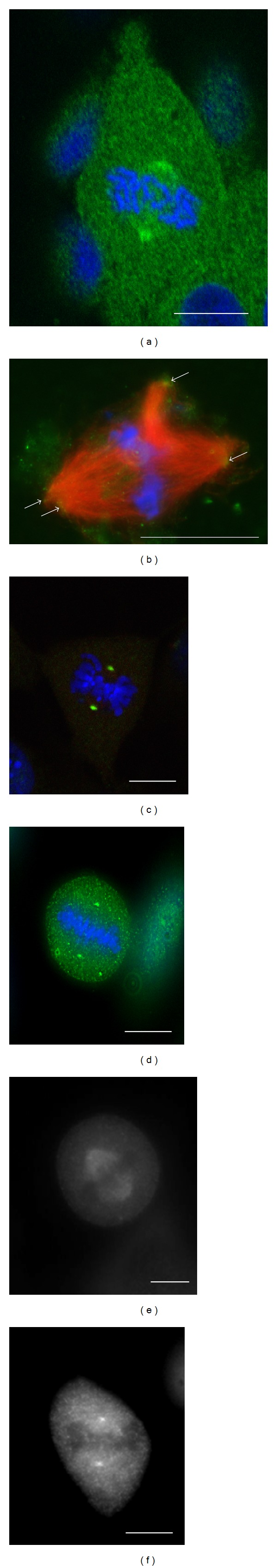
Immunofluorescence localization of TCTP in *Xenopus laevis* mitotic spindles using XlTCTP antibody. (a) Confocal section of an XL2 cell showing the presence of TCTP in the spindle with higher density at the spindle poles. (b) Isolated spindle formed by sperm-head addition to the CSF extract. Red MTs stained with rhodamine-*β*-tubulin added to the extract, green TCTP detected by immunofluorescence with XlTCTP antibody. White arrows point to spindle poles with TCTP staining. Blue DNA stained with DAPI. Note the presence of yellow staining of TCTP at the spindle poles and the absence of TCTP in the remaining parts of the spindle. (c) Confocal section of murine metaphase NIH3T3 cell stained with XlTCTP antibody (green) and with DAPI for DNA. Note that XlTCTP stains exclusively two distinct spots corresponding to the centrosomes, at the spindle poles corresponding. (d) Confocal section of human HeLa metaphase cell. Green TCTP detected with XlTCTP antibody, blue DNA. XlTCTP stains two spindle poles; the granular background staining is also visible in the cytoplasm. (e) Human HeLa metaphase cell. Green TCTP detected with homologous HsTCTP antibody, blue DNA. HsTCTP stains the whole spindle. (f) Monkey Cos7 metaphase cell incubated with anti-rat TCTP antibody showing a very distinct staining of spindle poles. Bar is equal to 20 *μ*m.

We focused our analysis on the localization of TCTP within the mitotic spindle because it allowed us to study simultaneously the association of TCTP with MTs and with centrosomes, which are located at the spindle poles. Immunolocalization of TCTP in mitotic *Xenopus laevis* XL2 cells clearly showed the presence of TCTP in the mitotic spindle with higher concentration at the spindle poles (Figure 1(a)). The spindle pole accumulation of TCTP was also evident in the spindles isolated from M-phase-arrested cell-free extract (Figure 1(b)). Because in mitotic *Xenopus laevis* cells and cell-free extract, the TCTP is associated with the spindle poles where the centrosomes are located, this suggests that TCTP may be a centrosomal protein.

TCTP is very evolutionary conserved protein [11]. Thus, we tested antibodies directed against different species TCTP in *Xenopus laevis* and mammalian cells. Surprisingly, when we used our polyclonal antibody against *Xenopus laevis* TCTP (XlTCTP) for TCTP detection in murine NIH3T3 and human origin HeLa cells (so called heterologous or interspecies detection), we always observed a very bright staining of centrosomes at the spindle poles (Figures 1(c) and 1(d)). However, when we used homologous antibodies and cells, that is, anti-human TCTP antibody to detect TCTP in human HeLa cells, a uniform staining of the whole spindle was visible (Figure 1(e)), which agreed with our previous study [5] and studies by Gachet and colleagues [12]. When we used another heterologous combination, that is, the-anti-rat TCTP antibody in monkey Cos7 cells, we also detected clear centrosomal staining (Figure 1(f)). These observations suggest that the subpopulations of immunologically distinct TCTP might be present in the mitotic centrosomes of human and monkey cells, similarly as in *Xenopus laevis *cells.

**Figure 2:**
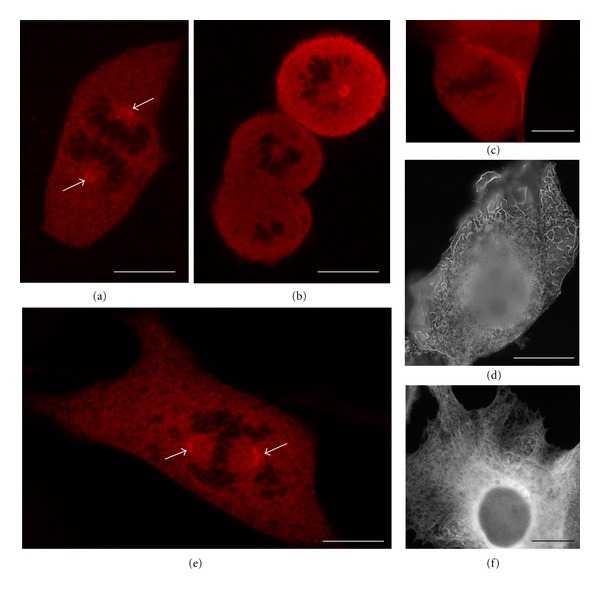
Expression of Myc-XlTCTP in *Xenopus laevis* XL2 cells. (a) Confocal section of XL2 cell in anaphase with high concentration of XlTCTP at the spindle poles (white arrows). (b) Confocal section of two dividing XL2 cells with high concentration of XlTCTP at the spindle poles. (c) Control mitotic XL2 cells expressing Myc tag only. (d) Interphase XL2 cell expressing Myc-XlTCTP. XLTCTP is localized in distinct fibers in the cytoplasm. (e) Mitotic murine NIHT3T cell expressing Myc-XlTCTP. High concentration of XlTCTP is present at the spindle poles (white arrows). (f) Interphase murine NIH3T3 cells expressing Myc-XlTCTP. Note that XlTCTP forms MT-like fibers in the cytoplasm. Bar is equal to 20 *μ*m.

To further clarify these observations, we expressed Myc-tagged XlTCTP in *Xenopus laevis* XL2 cells (homologous expression) and in mouse NIH3T3 cells (heterologous expression) and followed the localization of the recombinant frog protein in these two types of cells via immunofluorescence with anti-myc antibody. Figures 2(a) and 2(b) show examples of anti-Myc immunodetection of exogenous XlTCTP in XL2 cells. In these cells, we always observed MT-associated localization and an accumulation of Myc-tagged XlTCTP around a small negative area at the very tip of the spindle (Figures 2(a) and 2(b)). The control cells expressing Myc tag alone were uniformly stained (Figure 2(c)). In addition, in the interphase XL2 cells, the Myc-XlTCTP was incorporated into distinct cytoplasmic fibers (Figure 2(d)). The Myc-XlTCTP expression in murine NIH3T3 cells resulted in strong localization of TCTP to the spindle poles; however, we have never observed the presence of the TCTP-negative area similar to the one visible in XL2 cells (Figure 1(e)). In the interphase NIH3T3 cells expressing Myc-XlTCTP the frog TCTP was incorporated to the MT-like fibers (Figure 2(f)). These results show that TCTP indeed localizes to the spindle poles both in *Xenopus laevis* and in mouse cells, but the pattern of its localization is slightly different when homologous and heterologous system of immunodetection is used. Thus, exogenous Myc-XlTCTP is incorporated to the pericentrosomal area in the mitotic XL2 cells, while in the mitotic mouse cells, it is incorporated into the whole mitotic centrosomes. On the other hand, the homogenous immunofluorescence staining of XlTCTP visible in the spindle poles of XL2 cells suggests the presence of XlTCTP within the whole centrosomes. This indicates that, depending on the species or the cell type, the TCTP is localized either at the spindle pole within the centrosome or around the centrosome in the pericentriolar material (PCM) composed of specific proteins (including *γ*-tubulin).

**Figure 3:**
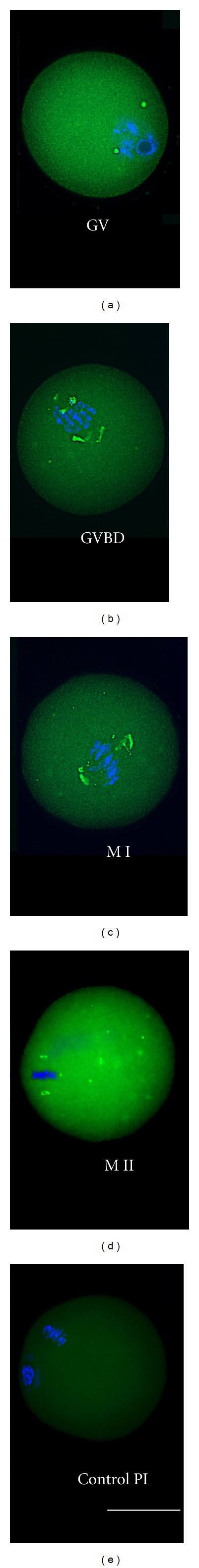
Immunofluorescence localization of TCTP with anti-XlTCTP antibody in mouse maturing oocytes. GV: prophase I-arrested oocyte, GVBD: the beginning of maturation, MI and MII: oocytes in MI and MII phase of meiosis, respectively, control PI: control MII oocyte stained with the preimmune serum. XlTCTP antibody stains PCM in all stages of maturing mouse oocytes. DNA (blue) stained with DAPI. Bar is equal to 40 *μ*m.

Mouse oocytes have no centrioles [13, 14], but they have irregular foci of PCM at the spindle poles both in MI and MII phases of meiosis [15–17]. Because mouse oocyte have PCM but do not have centrioles, we used maturing mouse oocytes to analyze whether TCTP associates with the PCM foci. When we stained *in vitro* maturing mouse oocytes with the anti-XlTCTP, we detected typical images of PCM foci (Figure 3) instead of the whole spindle staining observed when anti-rabbit TCTP antibody was used on mouse oocytes [18]. In GV stage oocytes arrested in prophase of the first meiotic division, a few distinct foci may be detected which are localized mainly next to the oocyte nucleus (called GV for Germinal Vesicle; Figure 3(a)), thus showing the number and pattern of distribution typical to PCM [19]. After GVBD (germinal vesicle breakdown) and during MI and MII, the TCTP-positive foci polarize at the relatively broad spindle poles (Figures 3(b), 3(c), and 3(d)). The same polarization of the PCM foci was shown by Schatten et al. [16], and Maro et al. [17]. Taken together, these results indicate that the subpopulation of TCTP detected by anti-Xenopus TCTP antibody indeed localizes to the PCM foci. 

**Figure 4:**
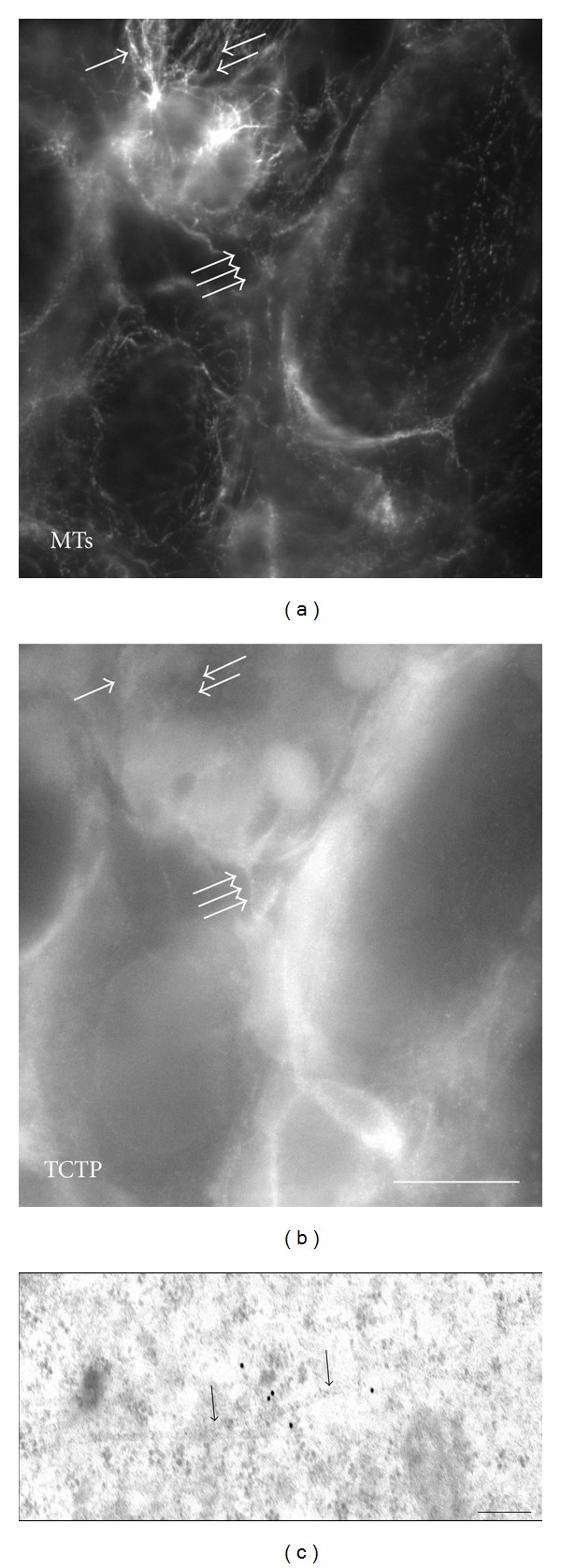
MTs and TCTP in *Xenopus laevis* tadpole ovary. Interphase cells. Upper panel, left: *β*-tubulin, right: TCTP localization. Anti-XlTCTP was used for this localization. Single white arrow points to cellular structures positive both for *β*-tubulin and TCTP. Double arrows point to *β*-tubulin-positive and TCTP-negative fibers. Triple arrows point to TCTP-positive and *β*-tubulin-negative fibers. Bar is equal to 20 *μ*m; Bottom panel: electron microscopy gold immunolabeling of TCTP (black particles in the center) in the vicinity of MTs (black arrows). Bar is equal to 100 nm.

**Figure 5:**
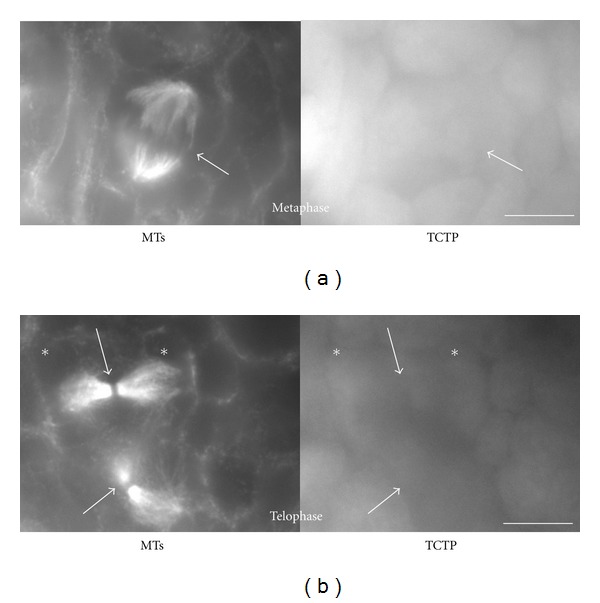
MTs and TCTP in mitotic *Xenopus laevis* oogonia. Left: *β*-tubulin, right: TCTP localization in tadpole oogonia. Anti-XlTCTP was used for this localization. Upper panel: metaphase cell. Left: mitotic spindle visualized by *β*-tubulin staining (white arrow). Right: The whole of the spindle is positive for TCTP (white arrow). Bottom panel: two telophase oogonia. Left: prominent midbides are visualized by anti-*β*-tubulin immunoflorescence (white arrows). White asterisks show the position of two daughter cells. Note the absence of TCTP in the midbodies. Bar is equal to 20 *μ*m.

**Figure 6:**
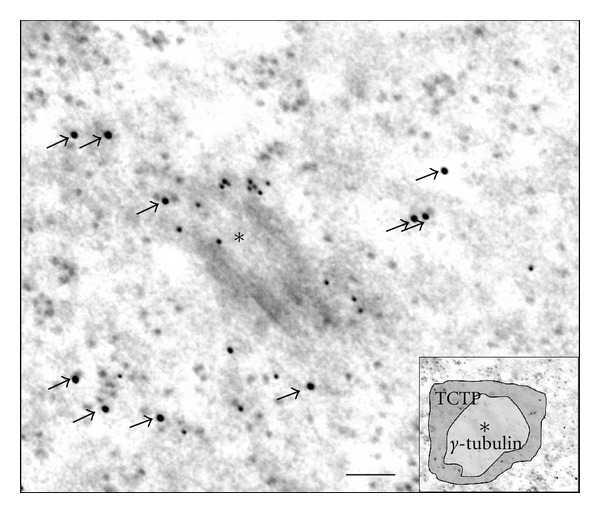
Double labeling of *γ*-tubulin and TCTP in the centrosome of *Xenopus laevis* oogonium. Centriole labeled with black asterisk; 18 nm gold particles (black arrows) correspond to the presence of TCTP, small, and 10 nm gold particles around the centriole mark the presence of *γ*-tubulin. Inset in the bottom right corner shows the central area around the centriole where *γ*-tubulin is present (clear central area), and the TCTP-containing external area of the centrosome (dark grey). Bar is equal to 100 nm.

In contrast to mouse oocytes, *Xenopus laevis* oogonia (or nest cells) have typical centrosomes formed by centrioles and the PCM [9]. We used these cells to analyze TCTP localization in relation to the MTs and centrosomes using light microscopy immunofluorescence and immunogold electron microscopy detection. Immunofluorescence using anti-*β*-tubulin and anti-TCTP antibodies in nest cells showed that the distribution of these two proteins was similar to their distribution in XL2 cells, that is, in the majority of cases these two proteins colocalized, but a subpopulation of MTs devoid of TCTP was also detected, and some TCTP-rich areas were devoid of *β*-tubulin (Figures 4(a) and 4(b); see [5] for details of similar localization of TCTP and MTs in XL2 cells). Electron microscopy immunogold labeling with the anti-XlTCTP antibody showed that TCTP was always localized at a distance of approximately 24 nm (the diameter of a MT) from the MT, but never directly on the MTs (Figure 4(c)). This indicates that TCTP does not associate with MTs directly, but by some intermediates serving as the linkers. Immunolocalization of *β*-tubulin and TCTP in mitotic *Xenopus laevis* oogonia showed that in the metaphase, the whole spindle area (detected with anti-*β*-tubulin antibody) was heavily stained (Figure 5(a)), while in the telophase, the tubulin-positive midbodies were negative for TCTP (Figure 5(b)) as already shown before in *Xenopus laevis* XL2 cells [5]. To facilitate identification of centrosomes at the electron microscopy level and to identify precisely the areas of the PCM, we detected anti-*γ*-tubulin antibody with secondary antibody conjugated with 10 nm gold particles and the anti-XlTCTP antibody with the secondary antibody conjugated with 18 nm gold particles. This double immunostaining showed that *γ*-tubulin is present in close proximity of the centriole within an irregular PCM cloud, and that TCTP is present in a layer surrounding the PCM (Figure 6, the inset in the bottom right shows schematically the distribution of *γ*-tubulin and TCTP domains around the centriole labeled with asterisk). Thus, the TCTP associates with the PCM of the centrosome, but it does not colocalize with *γ*-tubulin.

In conclusion, we show here that TCTP associates with the centrosomes in *Xenopus laevis*, human, monkey, and mouse cells and with the PCM foci in acentriolar mouse oocytes. Moreover, within the centrosomes, the TCTP associates with the external part of the PMC foci but not directly with the centrioles. We also show that TCTP associates with MTs at a distance of about 24 nm. This strongly suggests that the MT-TCTP association requires linkers, whose nature, at present, remains unknown. Though we still do not know the role of TCTP at the centrosomes, considering the fact that the aberrant duplication of centrosomes is a key factor in carcinogenesis (reviewed by [20, 21]), our observations open a new avenue into the study of TCTP/centrosome interactions. Interestingly, p53 was also shown to be associated with the centrosomes [22]. Taking into account the reciprocal negative feedback between TCTP and p53 [4], the potential role of TCTP within the centrosome may involve the antagonistic interaction between these two proteins.
